# Experiences of neurodivergent students in graduate STEM programs

**DOI:** 10.3389/fpsyg.2023.1149068

**Published:** 2023-06-15

**Authors:** Connie Mosher Syharat, Alexandra Hain, Arash E. Zaghi, Rachael Gabriel, Catherine G. P. Berdanier

**Affiliations:** ^1^Department of Civil and Environmental Engineering, University of Connecticut, Storrs, CT, United States; ^2^Department of Curriculum and Instruction, University of Connecticut, Storrs, CT, United States; ^3^Department of Mechanical Engineering, Pennsylvania State University, University Park, PA, United States

**Keywords:** neurodiversity, ADHD, autism, burnout, graduate education, STEM, masking, invisibility

## Abstract

**Introduction:**

Despite efforts to increase the participation of marginalized students in Science, Technology, Engineering, and Mathematics (STEM), neurodivergent students have remained underrepresented and underserved in STEM graduate programs. This qualitative study aims to increase understanding of the experiences of neurodivergent graduate students pursuing advanced degrees in STEM. In this analysis, we consider how common graduate school experiences interface with the invisibility of neurological diversity, thus contributing to a set of unique challenges experienced by neurodivergent students.

**Materials and methods:**

In this qualitative study, 10 focus group sessions were conducted to examine the experiences of 18 students who identify as neurodivergent in graduate STEM programs at a large, research-intensive (R1) university. We used thematic analysis of the transcripts from these focus groups to identify three overarching themes within the data.

**Results:**

The findings are presented through a novel model for understanding neurodivergent graduate STEM student experiences. The findings suggest that students who identify as neurodivergent feel pressure to conform to perceived neurotypical norms to avoid negative perceptions. They also may self-silence to maintain stability within the advisor-advisee relationship. The stigma associated with disability labels contributes a heavy cognitive and emotional load as students work to mask neurodiversity-related traits, navigate decisions about disclosure of their neurodivergence, and ultimately, experience significant mental health challenges and burnout. Despite these many challenges, the neurodivergent graduate students in this study perceived aspects of their neurodivergence as a strength.

**Discussion:**

The findings may have implications for current and future graduate students, for graduate advisors who may or may not be aware of their students’ neurodivergence, and for program administrators who influence policies that impact the wellbeing and productivity of neurodivergent students.

## Highlights

- During analysis, three overlapping themes emerged related to the unique experiences of neurodivergent graduate students: Internalization of Neurotypical Norms, Self-silencing to Make it Through Graduate School, and Neurodivergent Burnout Due to Overwork and Masking.
- The findings should be considered in the context of higher education, and the assumptions that are encoded within the institution. This includes beliefs and assumptions about what makes a “good” graduate student, the policies and power dynamics of higher education, and the advisor-advisee relationship.
- Neurodivergent students feel pressure to conform to perceived neurotypical norms to avoid negative perceptions and maintain stability within the advisor-advisee relationship.
- As neurodiversity is invisible, students may self-silence and mask their neurodiversity to survive the graduate school experience, as they fear that if deficit-based assumptions were applied to them, they might be perceived as less capable and thus miss out on financial support and career opportunities.
- The additional stress related to overwork and masking their neurodiversity may contribute to significant mental health challenges including increased anxiety, depression, and neurodivergent burnout.
- Despite the challenges that they face, neurodivergent graduate students perceive strengths related to their neurodivergence that may offer benefits to their graduate STEM programs.
- Graduate program administrators are in a position to provide faculty development to increase awareness of these challenges and build in policies that provide needed flexibility to support neurodivergent graduate students.
- Additional studies are needed to understand how the intersection of neurodiversity with other underrepresented identities including gender and race impact the graduate student experience for STEM students.

## 1. Introduction

The term neurodiversity encompasses a range of neurological variations such as, attention deficit hyperactivity disorder (ADHD), autism spectrum disorder (ASD), dyslexia, dysgraphia, dyscalculia, and other learning differences that are widely labeled and understood as disabilities (Armstrong, 2017; Haney, 2018). However, a growing body of literature suggests that many neurodivergent individuals possess traits such as divergent thinking, risk-taking, creativity, or spatial visualization skills (Hain et al., 2018; Syharat et al., 2020; Taylor et al., 2020a,b; Taylor and Zaghi, 2021) that may be assets in STEM fields. For example, divergent thinking and risk-taking have been correlated with ADHD (White and Shah, 2011; Taylor et al., 2020b), three-dimensional visualization skills have been linked to dyslexia (Attree et al., 2009; Rappolt-Schlichtmann et al., 2018), and pattern identification and systemizing abilities have been associated with autism spectrum disorders (Mottron, 2011; Crespi, 2021). Despite the potential of neurodivergent students to leverage these assets to contribute to innovation in their fields, they face a multitude of barriers and difficulties while navigating a traditionally rigid academic environment that demands strong skills across the board and encompasses expectations that students follow traditional approaches to problem solving, as well as negative attitudes and stigma (Clouder et al., 2020). These barriers often impede neurodivergent students from pursuing advanced degrees, thus depriving STEM fields of the skills of this talent pool.

## 2. Literature review

Despite efforts to increase the participation of marginalized students in STEM, neurodivergent students have remained underrepresented and underserved in STEM graduate programs (Honken and Ralston, 2013). This is evident by their high rates of departure from college (Honken and Ralston, 2013), lower-than-average levels of education (Kuriyan et al., 2013) and range of socio-economic challenges (Biederman and Faraone, 2006; Kuriyan et al., 2013) despite their comparable intellectual capabilities with neurotypical individuals (Kaplan et al., 2000; Weyandt et al., 2002; Sharp et al., 2003; Bridgett and Walker, 2006; Advokat et al., 2007; Jepsen et al., 2009; Taylor et al., 2020a,b). While accommodations may help neurodivergent students succeed in higher education, many choose not to disclose their diagnosis or seek supports from their university’s center for students with disabilities because they fear the stereotypes and stigma related to disability labels (Cortiella and Horowitz, 2014). For example, in one sample of engineering students who were formally diagnosed with ADHD, only 16.6% were receiving support services from the university (Zaghi et al., 2016). Further, graduate students tend to rely less on accessibility services as compared to undergraduates (Teichman, 2010) potentially because they are unsure if accommodations, which are most often geared toward the coursework needs of undergraduates, will be meaningful (Hain et al., 2018; Taylor et al., 2020a). From a research perspective, studies focused on neurodivergent students are found within the wider body of literature on students with disabilities, which makes it difficult to understand the unique experiences of neurodivergent students. The category of students with disabilities in STEM include students with visual disabilities, hearing disabilities, and other physical disabilities, while students with “cognitive disabilities” of any type are also often grouped together. For example, one report states that 7% of recipients of Science and Engineering doctoral degrees in 2014 reported having a disability; within this 7, 40% reported a cognitive disability, however, it does not specify which of these might be considered neurodivergent (National Academies of Sciences, Engineering, and Medicine et al., 2018). In addition, available research literature focuses largely on undergraduate rather than graduate students. Thus, the literature about neurodivergent graduate students within the context of STEM education is limited.

While the general undergraduate student experience of students in STEM has been widely studied over the previous decade (Litzler et al., 2014; Wilson et al., 2015; National Academies of Sciences, Engineering, and Medicine, 2017; Stanford et al., 2017; Craig et al., 2018; Fisher et al., 2020; Greaves et al., 2021), similar attention has not been paid to the graduate student experience (Satterfield et al., 2018; Berdanier et al., 2020). Graduate students face a unique set of challenges when compared to undergraduate students, including pressure to publish, financial insecurity, a highly competitive academic job market, work-life balance, and hierarchical faculty-student relationships (Wyatt and Oswalt, 2013; Slatkoff et al., 2016; Levecque et al., 2017); lack of transparency about university process; workload; role conflict (Mackie and Bates, 2019); the political landscape; and impostor syndrome (Woolston, 2017). Satterfield et al. (2018) noted that the pool of research on the graduate student experience in STEM was limited and compiled a comprehensive literature review in 2018 to set the stage for future work. This summary focused on the experiences of graduate students during their studies and explored how individual factors (the influence of the student’s advisor), programmatic factors (isolation and teaching assistantships), and external factors (work-life balance and family influence) influenced the persistence of graduate students in their field (Satterfield et al., 2018). Since then, Berdanier et al.’s (2020) study of social media forums found that among the factors influencing attrition in graduate engineering programs were the student’s advisor, support network, and goals, the quality of their life and work, and students’ perceptions of both program cost and how others perceive them. Several of these studies are limited in scope, in that they are focused on experience within a certain field, or department, such as chemistry or physics (Sachmpazidi and Henderson, 2021; Stockard et al., 2021). While these studies are valuable, we posit that the experiences of neurodivergent students in STEM disciplines may be different than those of the (likely) neurotypical populations described in these studies. While they may be experiencing similar challenges, the research community needs to specifically understand whether and how the worldviews and experiences of neurodivergent students may be different.

To that end, this research study was conducted to answer the following research questions: (1) What are the unique experiences of neurodivergent graduate students in STEM fields?; (2) How do neurodivergent graduate students in STEM perceive the challenges they face in graduate programs and how can/do they overcome them?; (3) How do neurodivergent graduate students in STEM effectively use their strengths to enhance their performance in their program?; and (4) How can this information can be effectively communicated with graduate advisors? This qualitative study uses thematic analysis to examine the experiences of 18 neurodivergent students’ in graduate STEM programs at a large, R1 university. We hope the findings inform changes in individual advising styles as well as broader programmatic structures. Likewise, we hope that additional research and empirical data related to neurodiversity in higher education may contribute to a culture shift in which the focus is on welcoming and cultivating the diverse cognitive abilities of students rather than on blaming individuals for their deficiencies.

In the sections that follow, we outline our theoretical frameworks and positionality in relation to the research. We then present an overview of the project, our study participants, and the research methods. These are followed by a presentation of the findings, a discussion of the findings in relation to existing literature, and the limitations of the study. We then provide a discussion of implications for research and practice. The paper concludes with a summary of key findings.

## 3. Theoretical frameworks

We frame neurological variations as an important facet of human diversity that may enhance society’s ability to address complex problems within STEM fields. Taylor et al. (2022) theory of complementary cognition suggests that cognitive diversity may strengthen the adaptability of human societies by making use of complementary cognitive strategies that balance societal needs for safety and risk-taking. Likewise, (Chapman, 2021) ecological model of mental functioning considers how individuals’ neurocognitive variations contribute to human ecosystems to support persistence and adaptation. This approach provides a framework for viewing neurodiversity as an integral part of human adaptation and suggests that the inclusion of neurodivergent individuals in STEM fields may enhance our collective potential for innovation for the benefit of society (Chrysochoou et al., 2022). We also take a strengths-based approach that emphasizes the assets related to neurodiversity, while acknowledging individual challenges and questioning the rigid conceptualizations of “normality” (Brown et al., 2021). The focus of the research is to enhance understanding of the challenges faced by neurodivergent students in graduate program environments, but also to contribute to understanding of their unique strengths and the ways in which they may thrive in graduate programs.

## 4. Researcher perspectives/positionality

Before discussing the results of this study, we would like to acknowledge our positionality in relation to this work. Our motivation and approach to this work is shaped by the personal experiences of several authors with ADHD and/or dyslexia, as well as our experiences working with a wide range of neurodivergent students within the context of neurodiversity-centered engineering and STEM education research projects. Our own experiences have led us to take a strengths-based approach toward neurodiversity that is integrated into the study, for example through the use of affirming language in recruitment and in our interactions with study participants. We believe that our shared experiences helped us to build a sense of rapport that opened a safe space for neurodivergent graduate students to share their lived experiences. We also believe it is important to acknowledge that while our team does represent diverse perspectives in terms of gender, cultural background, and other social identities, our perspectives are informed, and in some ways limited, by our experiences as white individuals in the United States.

## 5. Materials and methods

### 5.1. Project overview

This IRB-approved, NSF-funded research project included ten focus groups of graduate students in STEM disciplines at an R1 university in the Northeastern United States who self-identified as neurodivergent. Recruitment took place via an email that was shared through a listserv for all graduate students and an email from the university’s disability services office. The focus group participants (a) self-identified as neurodivergent, and (b) indicated that they were completing a graduate degree in a STEM field. Degree programs were classified as STEM programs based on the university’s list of STEM majors and/or their inclusion on the list of National Science Foundation Research Areas (NSF, 2022). Two participants pursuing STEM-related fields in the School of Education and the School of Business were also included. The students were pursuing a variety of majors across STEM fields within the College of Agriculture, Health & Natural Resources, the College of Liberal Arts & Sciences, the School of Business, the School of Education, and the School of Engineering.

### 5.2. Participants

Three rounds of focus groups were conducted to explore the experiences of neurodivergent students in graduate STEM programs. The ten focus groups included 25 participants. 7 of the participants who self-identified as neurodivergent reported only a condition [such as anxiety, depression, migraine, or post-traumatic stress disorder (PTSD)] that may or may not be related to an undiagnosed neurodiverse condition. These participants were included in the focus groups so as not to exclude individuals who may have not received a neurodiversity diagnosis, but who nonetheless identify as neurodivergent and perceive that their experiences fall under the neurodiversity umbrella. These 7 participants have been removed from the data set for the purposes of this analysis, which focuses on the experiences of individuals with neurological variations that may be clearly characterized as life-long conditions rather than conditions that may be acquired. The 18 neurodivergent graduate students in this data set took part in at least one focus group, with 4 of these individuals participating in two focus groups. Those participants who participated in more than one focus group responded to separate recruitment emails as part of separate focus group rounds, each of which explored slightly different topics related to experiences of neurodivergent graduate STEM programs. The presence of some individuals in multiple groups provided a level of nuance and depth to our understanding of the students’ experiences. If there were multiple responses by the same participant about the same topic, effort was made to ensure that these responses were not weighted more heavily in the analysis.

The majority of the participants were white women pursuing doctoral degrees. The reasons for the high representation of white women in this study are unknown. Participants were asked to indicate with which neurodivergent groups they identified; responses were recorded via open text entry. Nearly three quarters of the participants in the data set (72.2%) reported ADHD and 5 participants (27.8%) self-identified as autistic. Additionally, 38.9% reported a mental health condition such as anxiety, depression, or post-traumatic stress disorder (PTSD) as one of their neurodivergent identities or conditions. While the focus of this analysis is on the experiences of those with life-long neurodivergent conditions, it is important to note that participants perceived their mental health conditions as neurological variations that fall under the neurodiversity umbrella. 8 of the 18 participants (44.4%), identified with more than one neurodivergent group or condition. It is common for neurodivergent conditions to co-occur (Rubinstein, 2009; Germano et al., 2010; Vetri, 2020). Demographic data for the 18 participants are summarized in Table 1.

**Table 1:** Summary of demographic information (Total *N* = 18).

School/College	*N* (%)
College of Agriculture, Health & Natural Resources	3 (16.7%)
College of Liberal Arts and Sciences	10 (55.56%)
School of Business	1 (5.6%)
School of Education	1 (5.6%)
School of Engineering	3 (16.7%)
Neurodiverse Identity or Condition Reported	
Anxiety	7 (38.9%)
Attention deficit hyperactivity disorder (ADHD)	13 (72.2%)
Auditory processing disorder	2 (11.1%)
Autism spectrum disorder (ASD)	5 (27.8%)
Bipolar disorder	1 (5.6%)
Depression	4 (22.2%)
PTSD (post-traumatic stress disorder)	2 (11.1%)
Gender Identity	
Woman	11 (61.1%)
Non-binary/Other (e.g., Demigender woman)	2 (11.1%)
Man	5 (27.8%)
Race/Ethnicity	
Hispanic or Latinx	1 (5.6%)
Multiracial/biracial	2 (11.1%)
White	15 (83.3%)
Graduate Program	
MS (Master’s degree)	5 (27.8%)
PhD (Doctoral degree)	13 (72.2%)

Eight participants (44.4%) reported multiple neurodivergent identities or conditions. Participants reported mental health conditions including anxiety, depression, and PTSD alongside neurodivergent conditions such as ADHD or autism.

### 5.3. Data collection

Three rounds of focus groups were conducted to explore the experiences of neurodivergent students in graduate STEM programs. The data from earlier focus groups informed the development of questions for subsequent rounds, as the research team identified areas of interest that merited further exploration (for example, rounds 2 and 3 of focus groups explored prior educational experiences, experiences with writing, and experiences with and perceptions of accommodations). The focus groups were scheduled by participant availability and ranged from 2 to 5 participants. Each focus group was guided by a semi-structured protocol of open-ended questions centered around the participants’ experiences as students who identify as neurodivergent in graduate STEM programs.

We found that the online format did not hinder interaction among the participants. In fact, we found that the online format provided more convenience for participants, which allowed for a high level of participation and engagement. Additionally, the interaction among the participants in the online focus groups facilitated the sharing of experiences, allowing participants to build on each other’s responses, which led to rich and meaningful discussions. The group dynamic also was important in providing a space in which participants benefitted from hearing the experiences of others, reducing the sense of isolation and the sense that they were alone in their struggles. Participants expressed gratitude for the research and shared that they had both enjoyed and learned from others through the focus group experience.

Sample questions include, “What has been your experience so far, as a student in your STEM graduate program?” and “What do you think someone needs to do to be successful in your graduate STEM program?” The three rounds of focus groups, their areas of focus, and sample questions are summarized in Table 2. All focus groups were held virtually, via Microsoft Teams, and the videos were recorded and transcribed using Otter.ai (2022). The transcripts were edited for accuracy and pseudonyms were provided for each participant.

**Table 2:** Summary of focus groups.

Round	Area of focus and sample questions	(N) Groups
Round 1	Strengths and challenges, graduate school experiences, strategies, inclusive environments Sample Questions: What has been your experience so far as a student in your STEM graduate program? Can you tell us about the strengths you bring to graduate study in a STEM field?	4
Round 2	Advisor-advisee relationship, graduate-level writing experiences, understandings of neurodiversity Sample Questions: How would you describe your experiences with writing in your graduate program? How do you understand yourself as a neurodivergent person?	2
Round 3	Current and past educational experiences, current and past writing experiences, accommodations Sample Questions: Overall, how would you describe your experiences in your current program? What types of accommodations have been particularly important or useful to you in your academic experience?	4

Data from earlier rounds were used to inform the development of questions for subsequent rounds. A separate recruitment was conducted for each round of focus groups. The number of focus groups was determined by the number of participants who responded to the recruitment email and the availability of the participants.

### 5.4. Methods for data analysis

Qualitative methods allow for systematically exploring “the inner experiences of participants” (Corbin and Strauss, 2015). In this study, we conducted a thematic analysis with a constructionist approach which understands participants’ realities as both socially constructed and subjective (Braun and Clarke, 2021). According to this perspective, knowledge is created through the interactions and experiences of individuals within their social and cultural contexts (Gergen, 2015). Thus, we aimed to understand the ways in which the study participants made sense of their experiences within the context of graduate STEM programs. We followed the phases of activity described by Braun and Clarke (2006): “(1) familiarizing yourself with your data, (2) generating initial codes, (3) searching for themes, (4) reviewing themes, (5) defining and naming themes, and (6) producing the report” (p. 87). In this way, the raw data was examined for patterns to be systematically categorized and developed into themes that connect to existing literature or suggest new findings.

To familiarize ourselves with the data, we read and re-read the transcripts, adopting a reflexive approach to acknowledge and address our own biases and preconceptions. As the research was exploring an area about which there is limited existing knowledge (the experiences of neurodivergent graduate students in STEM programs) the initial codes were developed using an inductive coding process, allowing patterns to emerge naturally and intuitively from the data. Two researchers coded independently and then met to review codes collaboratively in order to ensure agreement about understanding of the data. Latent coding was employed to allow the research team to delve deeper into the underlying meanings and assumptions within the data. The initial list of codes was examined to identify and combine redundant codes. The remaining codes were then grouped into six initial categories.

The initial six categories and examples from the data were presented to a group of external experts who were engaged in the project as members of the advisory board. This group was comprised of university faculty who hold expertise in the fields of graduate education, educational psychology, engineering education, neurodevelopmental disabilities, twice exceptionality, and disability studies. Expert feedback was provided to the research team, who then engaged in an iterative cycle of analysis with these six categories, and three larger, interlocking themes were identified.

A preliminary visual map of these interlocking themes was then developed by the first author to organize, and visually depict the themes identified in the data. This preliminary map was then refined along with the co-authors to explore the dimensionality within the themes, identify areas of overlap, and explore the relationships between the three themes. For example, as part of this process the team probed the Neurodivergent Burnout theme for nuance, identifying overwork and masking as contributing to neurodivergent experiences of burnout. Additional graphic elements (overarching/surrounding circles) were also added to the visual map as part of this process to emphasize the contextualized nature of these experiences. This refined thematic map is presented as a novel model for understanding the experiences of neurodivergent graduate students in STEM.

## 6. Findings

**Figure 1:**
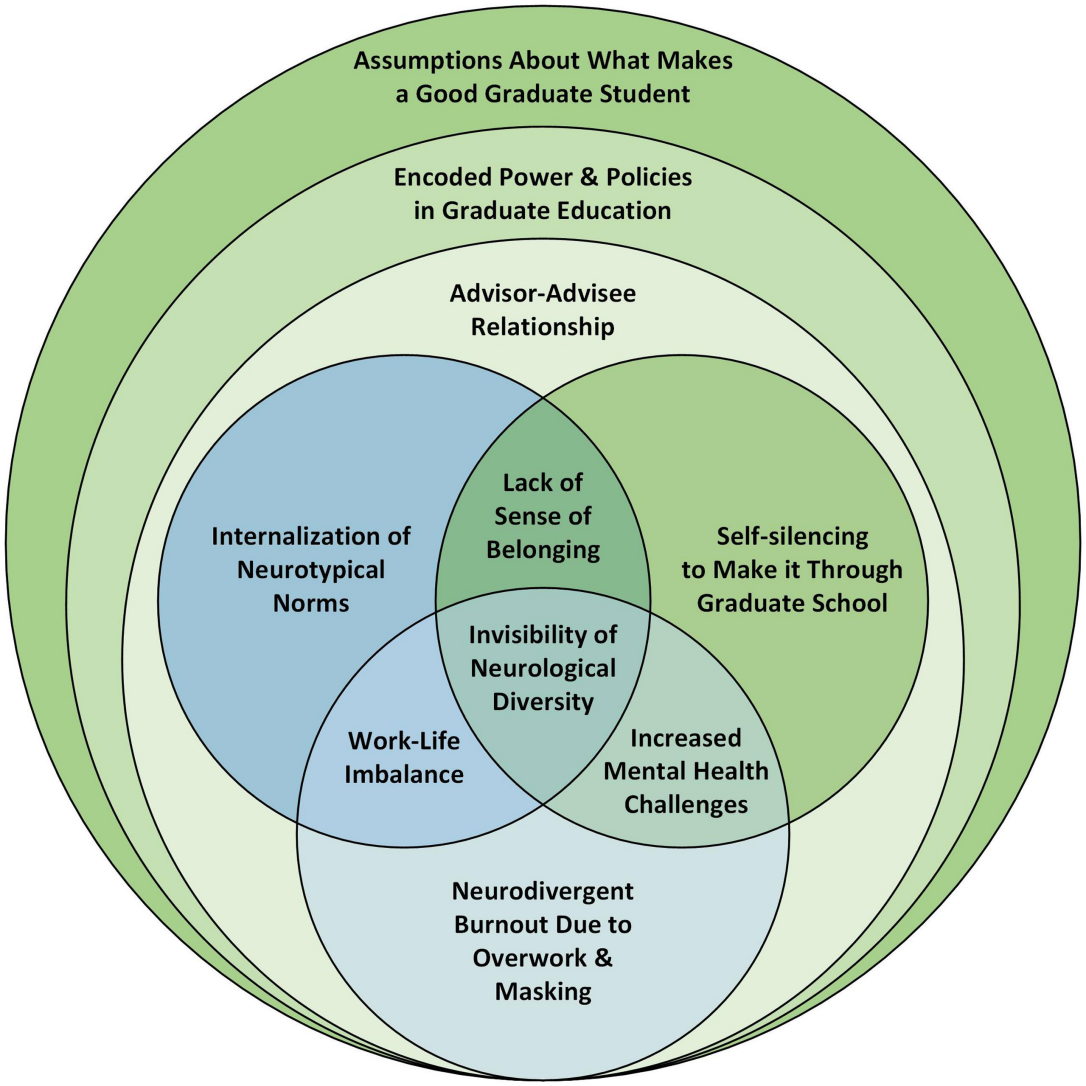
Thematic map presented as a novel model of the experiences of neurodivergent graduate students in STEM fields.

The findings from this study suggest a novel model for understanding the graduate school experiences of neurodivergent graduate students in STEM fields. This model places the invisibility of neurological diversity as a core feature of the neurodivergent student experience within the context of graduate STEM education. This model highlights how the invisibility of neurodiversity interfaces with common graduate school experiences and situates these experiences within overarching power dynamics that impact the wellbeing and productivity of neurodivergent graduate students. While some of the experiences described by the participants may be generalized to a wider range of students, such as graduate students with marginalized racial or gender identities, this model calls attention to a unique set of experiences that has not yet been represented in the literature about neurodivergent graduate students. This model, shown in Figure 1, is detailed below.

This model highlights three overlapping themes related to the unique experiences of neurodivergent graduate students: Internalization of Neurotypical Norms, Self-silencing to Make it Through Graduate School, and Neurodivergent Burnout Due to Overwork and Masking. These themes, along with relevant supporting data, are discussed in detail in the subsequent sections. At the intersection of these themes, the findings suggest that graduate students who identify as neurodivergent may experience a lack of sense of belonging, an imbalance between work demands and personal life, and the development of mental health challenges such as depression and anxiety. The fact that students’ neurodiversity is invisible to others in the graduate school environment unless they choose to disclose it may result in a dissonance between students’ sense of self and abilities and normative assumptions about who and what makes a good graduate student. The stigma associated with disability labels contributes a heavy cognitive and emotional load as students mask neurodivergent traits and navigate decisions about disclosure of their neurodiversity.

When considering the broader themes of higher education, it is important to understand the three overarching factors in Figure 1. These circles represent the assumptions of what makes a “good” graduate student, the policies and power dynamics of higher education, and the advisor-advisee relationship. Neurodivergent students are subject to systemic power dynamics and policies that privilege certain ways of thinking, learning, knowing, socializing, and being (Lynch and Macklin, 2020). Consequently, neurodivergent graduate students must navigate complex interpersonal dynamics that are heavily influenced by the hierarchies of academia, particularly in the advisor-advisee relationship. In this way, neurodivergent individuals may struggle to meet the expectations embedded in these circles of power and may find themselves marginalized and excluded from the higher education system.

### 6.1. Internalization of neurotypical norms

The students in this study reported that the ideal graduate student should possess attributes that they assumed were found in their neurotypical peers, such as strong executive functioning (i.e., skills including time management, goal setting, planning, and emotional regulation). Since autistic students and those with ADHD or dyslexia often have challenges in some of the areas in which they feel they are expected to possess strengths, they may struggle to feel that they belong. Alexis, went on to describe the institution as a sort of assembly line through which only certain types of students are allowed to pass, when she said:

…the program is a circle, like chute thing and like some of us are squares and they're trying to press the squares through a circular tube and like not all of us fit. And so sometimes we get left out of things. And we're not included in things because the program and the institution were not built for people like us in mind.

Multiple students suggested that advisors may contribute to expectations about who is a “good fit” for their program. For example, Ted says that “not every advisor knows… that people are different and cannot just like all work the same way. And then you know, maybe they do not care. Maybe they are just like, if you do not fit, you do not fit.” Meanwhile, Ted pointed out that some advisors may have expectations based on how they function or how things were when they were in graduate school, which promote conformity to neurotypical norms:

Like, you might say, ‘Oh, I will operate differently.’ And they say, ‘You can’t do that.’ And it’s like, well I can’t control that. So, now I’m just being punished for who I am.

Additionally, participants discussed graduate school expectations related to specific skills and behaviors, such as efficiency, strong reading, writing, and public speaking skills, the ability to process information quickly, and the capability to conform to expected norms related to schedules, timeliness, productivity, socializing, and communication. Often, the participants described a difference between their performance and the expectations for a graduate student and then expressed a negative self-judgment if their performance did not match the expectations. For example, Alexis, who identifies as autistic, described how the expectations that graduate students should be able to quickly process difficult questions contributed to her perception that there were “awkward” moments during presentations. In her words:

I love giving presentations, I like sharing my ideas about my research. The Q & A portion is where I struggle. Because I think I have this expectation for this idea that other people expect me to like, have a very fast processing speed and like, I can verbally say the things in like the perfect way, whenever they ask me a question. I know that was something that I struggled with when I was defending my master's thesis. And I feel like I didn't get my point across as well as I could have. If I like, had a little bit more time to think about it. Like I could have just like, sat there and paused. Like, that's kind of awkward and just like have people sit there while you're going through all your thoughts and like putting it into the package.

Similarly, Robin, describes how ADHD, anxiety, depression, and PTSD contribute to their need for significant time to rest after the workday and expressed negative self-assessment while comparing their performance to their neurotypical counterparts:

Yeah, I think the main thing that I struggle with is the self-deprecation and the comparison. Because I work in an office with a lot of neurotypicals, who just like can write and ponder about these things all day, and like, don't ever shut off… So listening to these people and be like, Why can't I be like them? Why can't I be like them? Why can't I be like them? And I'm like, I'm lucky if I get one good day, a week, let alone a month, where I can do something partially similar to what they're doing.

In one example, students shared how ADHD and anxiety shaped their struggles with getting started on a writing task and expressed harsh self-judgments about these challenges, describing their procrastination in both positive and negative terms. For example, Wendy says, “But yeah, I do not do anything until the very last minute ever. And then I just panic about it. And then it magically gets done. I have not figured out what the like magic part is yet,” and Moira states,

“I tell myself whenever I procrastinate, or whenever I'm like doing something late at night, like at the last minute, and like, it always gets done. I don't know how but it always gets done… I've never handed anything in late. So, it must just be working, which is really not a healthy mentality to have. But like, you know, like, but it gets finished.”

### 6.2. Self-silencing to make it through graduate school

We found that the neurodivergent students in this study exhibited aspects of self-silencing as a strategy for maintaining stability within their advisor-advisee relationship in the face of what they often perceived as a threatening power dynamic. For example, Alexis describes how neurodivergent students mute themselves, rather than engage in self-advocacy to seek support for their struggles, to get through their program:

I know many people just in my program who hide it, because people who do mention it to faculty members are treated much differently. And so, it’s kind of easier to just deal with it on your own and not tell anyone, and maybe struggle behind the scenes. But that’s still sometimes better than letting them know and having them treat you much worse or much differently.

As the students in our focus groups described their interactions with their advisors, they used language that showed awareness of the existing hierarchical power structures within their STEM programs and how these structures impacted their willingness to ask for help from their advisor. For example, Twyla specifically explores the impact of power dynamics on her readiness to communicate about her struggles, while also mentioning serving those who are “above” her in the hierarchy:

I feel like there, there's an expectation or… with power dynamics, like, you've kind of feel like you're below the administration, or somehow, you're serving the administration, or whoever you perceive is above you. And so, you need to present yourself a certain way. And that might not be their expectation, but I think I mean, my perception is that… how a professional person would be defined in our society, would be someone who doesn't really talk about personal things, or how they're struggling with their work. You know, you don't want to tell your boss that or someone above you that you're struggling with the work or the academia or what have you. And that's really difficult, especially because that's like, a personal barrier that you have to fight with, if you need help with something.

Alexis also shared how she often chose to silence herself about the challenges that she was facing because the faculty have power over her future. She describes this as follows:

I think for me, it's just, I dunno struggling with the idea of not maybe not being able to be as honest about difficulties within the program, either like, on a personal level, or just things I don't agree with, like at a departmental level, because like, faculty members have power over my career, and like have to write me letters for funding things, or, like if getting an internship or things like that… And so, sometimes I approach other people and like faculty members kind of in that same way, but like, I've had to adjust how I talk to people who have a higher status than me. Yeah, I think it's just understanding that I can't say certain things that I really wish that I could say because they have a hold on my career, and I want my degree and to do the things that I want to do.

Pleasing behavior, another aspect of self-silencing, is often related to negative self-judgments and a reliance on compliance with others to gain their approval (Baeza et al., 2022). In this study, students used communication aimed at avoiding conflict with or disapproval from their advisor. Students often chose to not ask for help and prioritized their advisor’s needs or feelings, often at their own expense. For example, Marnie avoided communicating with her advisor about her lack of steady progress on her project, ultimately completing a large amount of work in a short period of time in order to meet a deadline. In this way, she was able to maintain the approval of her advisor. She says:

But I also don’t want to bring something up that will make them think about me differently. Because I know that I am capable, when I’m on top of things. But when I’m not on top of things, I can be a mess. And that’s the reality that I know about myself, but don’t like to acknowledge. And so, like this past month, it’s been a lot more of like I’m trying to hide things, because I haven’t been on top of things. And so, like that whole, like six weeks’ worth of work that I should have done… I did in two days because I was meeting with him the next day, and I had to have stuff to show him. So, I stayed up all night to have stuff to show him because I didn’t want to bring up why I wasn’t getting things done with him in the first place.

Patrick also hesitates to blame his advisor for miscommunications, implying that she could not have communicated poorly, given her high status. He says:

But I was like, I misunderstand what I am expected to do, after I'm given instructions… And I've tried to evaluate whether that's, I guess, whether I'm more at fault, or my advisor's more at fault… And also, I know if it's like, kind of like a hierarchal perspective, like, because I think highly of her maybe it's like, I just can't grasp what I would normally be able to grasp from another person.

Other students showed patterns of communication in which they struggled to voice their experiences due to perceptions of power. For example, Twyla notes that, “it’s a little bit harder to be honest about your struggles, or, like your feelings about academia when there are people in higher levels around you,” and Marnie says, “I’m not good at having conversations with… authority figures talking about… feelings.”

### 6.3. Neurodivergent burnout due to overwork and masking

The students in our focus groups noted that the increase in workload as compared to that of undergraduate studies placed a high demand on their organizational skills and contributed to both high levels of stress and poor mental health. Participants noted that the difference in workload intensified some of their challenges with time management, motivation, and prioritization. For example, Gwen described how she struggled to adjust to the demand for independently getting started on her graduate work:

… at the beginning, I was very, very bad at and I've worked very hard to, like, be better at if that makes sense…. a lot of like, grad work is kind of independent. So like being able to start independent work, being like, I would be like, where do I start if I run into a problem? I'd be like, well, I guess I'm done. I don't know what to do.

Twyla noted that,

…in grad school, the work is not consistent. And so, like sometimes you have lulls. Sometimes you have deadlines happening all at the same time. And then, so, like, it’s hard to distribute your productivity evenly throughout your time… And also, my brain, and like, my brain can’t, like distribute all of these things evenly. And like, also, like prioritization, I think is another big thing just between life and work and all the responsibilities.

Similarly, Grace describes the process of learning to juggle the new responsibilities of her doctoral program while also expressing that she is not free to set boundaries that might allow her to limit the number of activities that she takes on:

And so yet, especially like, with graduate school, you end up juggling a lot of things. And I feel like I've started figuring out the balance. And then I officially joined a lab. And now it's different again. And I happened to join a lab where I'm the only PhD student. And so that's a whole ‘nother level of things like there's nobody else PhD student sort of rank… that's doing that with me. And so then, yeah, the balance is like, I don't know where I can say, I've got too much on my plate, because I'm the only person who can take care of a bunch of this stuff.

Wendy noted the heavy workload and how she often neglects self-care in order to meet the expectations of the program, but she also comments on the frustrations that she experienced trying to force herself to work “in regimented time,” like neurotypical peers. She says:

I will work until everything is done and everything is beautiful and wonderful. And all of the things have been squared away and put where they belong, and it's wonderful. And if I have to not sleep for three days to do that, that's what happens… I didn't get professionally diagnosed until I was 25, 26. So I went through high school and college really frustrated all the time. I couldn't understand why I couldn't just work in like regimented time.

Other factors may also influence neurodivergent students’ sense that they need to work harder and longer than other students. Several participants discussed experiencing imposter syndrome, which may be described as the feeling that one is a fraud despite one’s accomplishments (Meadhbh Murray et al., 2022). These experiences are described by Moira, who said:

But I think that it also has to do with… imposter syndrome and me thinking… I have to take on all this stuff to prove that I am… capable and able to do that, I'm competent and… can be successful. So, sometimes I'll overload myself because of that.

Marnie built on Moira’s statement, saying:

I would say the beginning was horrible. I… imposter syndrome. And like not knowing the name for imposter syndrome. I definitely went through a lot of depression. Also not knowing or ever thinking if I like had some sort of like cognitive behavioral thing. So probably that was affecting me in a way I didn't understand… And I, just like Moira, have trouble saying no to things.

Participants questioned their ability to manage their heavy workload while simultaneously perceiving that others do not struggle in the same way. For example, Jim wondered if “maybe it’s not that way for neurotypical students.” Alexis expressed the feeling that the graduate school workload may take a particularly heavy toll on neurodivergent students when she said:

…some people might be able to do that for long periods of time and like maybe not experience burnout, but then other people might experience burnout more quickly with those things… But I think there's this expectation of like, you should just do X, Y, and Z and overload yourself, and you'll be fine, because everyone else has been fine doing that. But that would take me out longer. And I would need a longer period for recovering from that.

These students perceived that others were not experiencing the same level of challenge in managing the demands of their program, and that navigating graduate school exacted a terrible toll on their energy that went beyond what neurotypical students might experience.

In addition to the students’ experiences of overwork, many described how they put energy and effort into masking their neurodivergence. Alexis describes this when she says,

And sometimes, like, professors don’t have the skills or the knowledge to understand our perspectives… So, like, sometimes, the burden is placed on students to kind of like dull their personality or like, be a certain way, just so they can make it through the program…

Meanwhile, Ted fears what will happen if he is open about his neurodivergence, which he identifies as ADHD, an anxiety disorder, and a depressive disorder. He says,

To me, it's like the threat of consequence, I guess in in being open or something, right?… Like, that kind of thing. I mean, I guess the upside is if they are open and accepting, and they're working with you, but I guess just I don't know, if you're weighing costs versus benefits. The… there's a big downside. And big upside, I guess. Oh, it's just like, a very intense situation.

Nancy, who reports an auditory processing disorder and an anxiety disorder, describes how daily efforts to mask her neurodivergence in front of her advisor result in increased anxiety about revealing parts of herself, when she says:

I do this masking where I put on that I'm very together for - in front of her and I have all these plans, and my calendar is all marked, but then my day to day, I don't feel like that. So, like revealing that side of me, is something that gives me anxiety.

### 6.4. A different perspective

While the students’ perceived strengths was not as extensive within the data set, and thus was not represented as part of the model presented in this paper, we believe it is important to note that alongside the tremendous challenges faced in the graduate school environment, many of the graduate students in this study expressed an appreciation of their neurodivergence and the unique approaches that they bring to their STEM field. Specifically, multiple participants mentioned that they bring different perspectives, creativity, and problem-solving abilities to their work. For example, Alexis says,

But I also think I just think of things and conceptualize things in a different way compared to other people. And so I might see patterns and things that people didn't see, which people have said has been helpful, like, I give a different perspective on something that people didn't think of before.

Similarly, Grace expresses enjoyment of her creativity, despite the frustrations that she experiences in some areas of her life. In her words:

I do remember being asked once that, like one of my psychiatric diagnosis things, one of the questions that the doctor asked was, do you wish that you didn't have it? And that's a very, pretty emphatic no, like, No, I wouldn't change that about myself. Because I appreciate too much the way my brain does think about things. But I'm still upset at the like, these things are like, I'm staring at my pile of laundry. And it's not magically getting itself done, kind of stuff, but I am too attached to, I think I'm really good at my creative like, uh… words. I like too much the way my brain thinks about things to want to change it. Even with all of the downsides to everything. I feel like it's informed too much of who I want to be and what I want to do with my career and my life and all of that.

In addition, several participants described strengths related to their work ethic, highlighting their ability to take on multiple tasks, even if it meant pushing themselves to their limits. For example, Ted describes how he leverages his interests for motivation in his work:

I guess like, if I'm motivated and interested, I can work very hard at something. And also, as far as just like absorbing information, I'm very good at that, and then making connections. So you know, as long as I find something interesting, I'm willing to work very hard at it.

Meanwhile, Wendy describes both her flexibility and tendency to work hard when she says:

I think I can figure out a way to do something, or do most anything. I guess that's a strength. So my advisor says, This has to get done. Whether it's design a massive experiment, or figure out how to hire people or conduct interviews, or write a paper in a week or do a grant. Like, I'll figure out how to do it, it may not be the healthiest way, but it'll get done. Um, so I guess how do I articulate that into a… reliability, I guess that's a strength. If I say I'm gonna do something, it'll get done. Some sometimes it'll get done damn the consequences, but it'll get done.

Finally, participants described their openness about their mental health and neurodiversity as a strength that both supported their own wellbeing and helped create a more inclusive environment for others. This is exemplified by Grace’s statement that:

I now talk openly about my mental health because other people did, and it helped me. So I think, I think that gives me a strength. And I'm trying to like continue on from what other people did for me.

## 7. Discussion

If we aim to enhance the learning environment for neurodivergent graduate students in STEM fields, it is vital that we first understand their experiences in STEM graduate programs. Since the majority of research centered on neurodivergent students is deficit-based and focused on undergraduate students, this study aimed to explore (1) the unique experiences of neurodivergent graduate students in STEM fields; (2) neurodivergent graduate students’ perceptions of the challenges they face in graduate STEM programs; (3) how neurodivergent graduate students in STEM use their strengths in the context of their program; and (4) how this information might be effectively communicated with graduate advisors. A discussion of the findings is presented in the following sections.

### 7.1. Internalization of neurotypical norms

Throughout, the students expressed frustration with themselves as they attempted to conform to the expectations that they will work like their neurotypical peers. For example, the participants suggest that procrastination, while stressful, triggers a state in which a task that has been avoided until the last minute can suddenly be completed in a short amount of time. Moira’s assessment of her work style as “an unhealthy mentality” points toward an internalization of norms that value steady, goal-oriented progress over bursts of productivity triggered by stress. Yet, both Moira and Wendy characterize procrastination as a sort of “magic,” that helps them get work done. We consider the possibility that, despite the anxiety and stress related to procrastination, it may be used as a tool by some neurodivergent students who, by waiting until the last minute, set off an internal process that fuels productivity.

The data pointed toward the internalization of the expectations of how a graduate student should work, as they voiced negative self-judgments when they deviated from these academic norms; students navigated choices related to openly sharing their struggles and thus making their neurodiversity visible or working extra hard in order to “fly under the radar.”

As Dolmage (2017) notes, “the ethic of higher education still encourages students and teachers alike to accentuate ability, valorize perfection, and stigmatize anything that hints at intellectual (or physical) weakness” (p. 3). This culture of perfectionism places pressure on students to dedicate all their waking hours to their work as they attempt to meet impossible expectations (Yu et al., 2016). These norms are particularly salient at the graduate level, where intellectual ability is of primary importance. These assumptions create an environment that holds up neurotypicality as the ideal and encourage neurodivergent students to hide any implications that they are deviating from these norms.

### 7.2. Power dynamics and self-silencing

Higher education is marked by highly stratified power structures based on positional power and access to resources within the university structure (Lee, 2022), as well as overarching and intersecting societal structures such as race, class, sexual orientation, gender, and ability (Bell et al., 2018). These societal and institutional structures contribute to power differentials that impact graduate students’ relationship with their advisor/supervisor, working conditions, mental health, and persistence in their degree program (Lee, 2022). These power dynamics place graduate students, particularly those with marginalized identities, in a vulnerable position in relation to their advisor’s policies, attitudes, assumptions, and practices. Since neurodiversity is an invisible aspect of diversity, faculty may not be aware of the ways in which power dynamics and assumptions impact their interactions with neurodivergent students.

While these power dynamics likely impact all graduate students to some extent, many of the graduate students in this study perceived that disclosure of their neurodivergent identity placed them at particularly high risk of poor treatment, negative consequences, and discrimination and particularly noted the impact on the advisor-advisee relationship and potential career outcomes. While the experiences of the individual students varied depending on the culture within their program and the attitudes of their faculty advisor, many students preferred to not share information about their neurodiversity with their advisor. The need for students to hide their neurodiversity was, in many ways, tied to the perceived possibility of negative financial and career outcomes if they disclosed. Many students made the choice to remain silent about an important piece of their identity and life experience in order to make it through the program.

Previous scholarship has used the concept of self-silencing to describe women who suppress their own voice in order to conform to societal expectations of femininity within heterosexual relationships, which may make them more vulnerable to depression (Jack, 1991). However, more recent work related to self-silencing has suggested that this concept may be applied more broadly across genders and populations to describe how self-repressive behaviors may serve the purpose of maintaining relationships, especially when these relationships are marked by power differentials (Baeza et al., 2022). Key characteristics of self-silencing include inhibition of self-expression, selfless behavior, and pleasing behavior (Baeza et al., 2022). Self-silencing has been found to be related to depression, psychological distress, low self-esteem, and reduced self-care (Baeza et al., 2022), and in an educational context, to feelings of disconnect from one’s teachers and hesitancy to seek help (Patrick et al., 2019). Patrick et al. (2019) wrote, “students faced with the perception of threatening power relationships, whether gender-based or otherwise, might be especially likely to be willing to sacrifice autonomy as an ill-fated strategy for preserving relatedness with the powerful other, the teacher” (p. 946). Our model builds on prior research related to self-silencing by highlighting the ways in which neurodivergent students self-silence in response to institutional hierarchies and interpersonal power dynamics within the graduate school setting.

Since most neurodivergent students consider accommodations to be related to coursework, they often do not think that relationships like the advisor-advisee relationship can be managed toward their success in graduate school While flexibility on the part of the advisor may support these students’ unique needs and ways of functioning, accommodations are insufficient to address neurodiversity within the context of the advisor-advisee relationship. An approach in which accommodations are offered by the advisor to mitigate student weaknesses both reinforces the power dynamics inherent in the advisor-advisee relationship and risks failing to recognize the strengths of neurodivergent students that might otherwise be cultivated and leveraged for the benefit of society. Advisors who are aware of their student’s neurodiversity-related strengths and challenges may work alongside their student to develop an understanding of the ways in which their student may best use their strengths within their graduate program. In contrast, neurodivergent students paired with an unaccommodating advisor may face distinct challenges as they navigate their program; their relative lack of power within this relationship coupled with their advisor’s rigid expectations and role as gatekeeper within their field means that neurodivergent graduate students may seek strategies that allow them to maintain a relationship with their advisor by staying silent about their neurodiversity, hiding their struggles, and masking their differences.

While other marginalized groups of students may have similar experiences, we suggest that neurodivergent students may be particularly vulnerable to the power dynamics embedded within graduate programs due to the assumptions related to intellectual ability within academia as well as the predominant perception of neurological diversity as cognitive impairment. Due to the invisibility of neurological diversity, students may be perceived as neurotypical if they do not disclose it. Thus, students may attempt to mask their neurodivergence to blend in with their peers. Many of the neurodivergent students in this study sensed that if deficit-based assumptions were applied to them, they might be perceived as less capable, lose funding or positions on research projects, or miss out on recommendations from faculty who are renowned experts in their field, and thus remained silent.

The communications between student and advisor presented here may be indicative of broader patterns among graduate students. However, we suggest that this dynamic is complicated by neurodivergent students’ perceptions that they need to silence their neurodiversity-related experiences to maintain stability in their advisor-advisee relationship and avoid negative consequences.

### 7.3. Neurodivergent burnout

The students in this study demonstrated a pattern in which they placed the highest importance on the needs of their advisor and project and worked long hours to meet the expectations placed on them by their program. Rather than prioritize their own needs for self-care and personal development, they often struggled to set boundaries with their advisor to maintain a healthy work-life balance. While this pattern of selfless behavior may also be a component of self-silencing, this is most concerning as it relates to neurodivergent burnout.

It is well known that graduate school is a high stress environment that places students at risk of burnout, which may be described as a “work-related syndrome resulting from chronic exposure to job stress” that is marked by “emotional exhaustion, cynicism and depersonalization, reduced professional efficacy and personal accomplishment” (De Hert, 2020, p. 171). Within STEM fields, this risk may be even greater, as students face tremendous pressure and are found to spend up to 80 h a week on their schoolwork, often at the expense of their own self-care needs (McDermott and Bahr, 2021). This high-stress environment likely has a disproportionately high impact on the wellbeing of neurodivergent graduate students, who may work long hours to compensate for challenges (such as differences in attention, time management, or reading ability), and also spend significant energy in masking their neurodivergent traits.

And even though many graduate students may experience imposter syndrome at some point in their career, these feelings likely place a heavy burden on neurodivergent graduate students who may feel pressure to hide their challenges to prove that they belong in academia. As Meadhbh Murray et al. (2022) write, “Students expend time and energy doing emotional work to navigate imposter feelings with marginalized students experiencing more persistent and intense imposter feelings than their more privileged peers, often in response to, and reinforced by, the exclusionary atmosphere of the university” (p. 2). This dynamic may lead some neurodivergent students to push themselves beyond what they believe is manageable to prove that they are capable, ultimately risking burnout and exhaustion.

The literature suggests that autistic individuals in particular may develop elevated rates of anxiety, burnout, and even suicidal ideation in relation to their experiences with masking to cope with stressful environments (i.e., environments designed around neurotypical ways of being) (Higgins et al., 2021; Radulski, 2022). Additionally, it has been suggested that ADHD and PTSD may be underlying factors related to the development of chronic anxiety, emotional exhaustion, and burnout in stressful work environments (Brattberg, 2006). The finding that a majority of students in this study described some element of hiding or masking their differences from others within the graduate environment, indicates that students who identify as neurodivergent may carry a hidden load as they expend significant mental and emotional energy to hide their neurodivergence, and thus may be at higher risk of burnout from the overwork of graduate school than neurotypical peers.

The findings of neurodivergent burnout in relation to overwork and masking in the graduate school environment is of particular interest, since masking is primarily discussed in the literature and online communities related to autism, and to a lesser extent in relation to ADHD (Hallberg et al., 2010; Fugate, 2014), and may be defined as covering or modifying one’s neurodivergent behaviors to blend in with neurotypical people (Radulski, 2022). Camouflaging to pass as neurotypical is associated with decreased mental health among neurodivergent individuals (Cage et al., 2018; Shmulsky et al., 2022).

Existing literature about the graduate student experience indicates rising rates of anxiety, depression, and burnout across the board (McDermott and Bahr, 2021; Meluch, 2021). Even prior to the COVID-19 pandemic, which has contributed to high levels of stress, anxiety, and depression among college students (Son et al., 2020; Council of Graduate Schools and The Jed Foundation, 2021), one study found that 41% of graduate students showed moderate to severe anxiety and 39% had moderate to severe depression (Evans, 2018). As our study participants’ experiences were recorded during the COVID-19 pandemic, it is possible that their mental health challenges may have been amplified by increased isolation and anxiety (Dragioti et al., 2022). The findings presented in this paper adds to the literature about graduate student mental health by showing that students who identify as neurodivergent face unique stressors in graduate school program environments that may exacerbate or contribute to the development of mental health challenges, including depression, anxiety, and burnout.

### 7.4. Perceived strengths

Despite the intense challenges faced by neurodivergent graduate students in STEM programs, many perceived strengths related to their neurodivergence. The fact that these graduate students perceived their neurodiversity as a benefit to their STEM program points to the need for additional research into the strengths of neurodivergent students. The literature related to neurodiversity has historically favored an overwhelmingly deficit-based perspective. Additional strengths-based literature has the potential to transform the way that neurodivergent individuals understand themselves and their neurodivergence while also contributing to a larger shift in the way that neurodiversity is perceived within higher education and society as a whole.

## 8. Limitations

Participant inclusion in the study was based on participant self-reports of neurodiversity and/or diagnoses; no formal measures were used to confirm self-reported diagnoses. While self-reports may yield some inaccuracies, no diagnostic process is entirely foolproof. For example, the literature related to ADHD and autism points to significant delays in diagnoses of women, as well as a tendency for women to present with anxiety or depression, while their ADHD or autism goes unrecognized (Quinn and Madhoo, 2014; Kentrou et al., 2019). Likewise, the current literature indicates significant disparities in diagnosis and services for neurodivergent individuals from racial or ethnic minorities (Zuckerman et al., 2014; Moody, 2016; Haack et al., 2018; Chen et al., 2019; Shmulsky et al., 2022). Thus, relying on diagnosis for inclusion in this study might further limit the participation of racially or ethnically marginalized students who also identify as neurodivergent. Another limitation is that the majority of participants were white female doctoral students, which may reduce our ability to understand how experiences of neurodiversity may vary across social groups and identities. For example, given the higher relative representation of women in this study, it is possible that the themes may be skewed toward the experiences of neurodivergent women in graduate STEM programs. The intersection of gender, race, and neurodiversity is outside the scope of this work, and should be further explored to gain insight into how these factors influence neurodivergent students’ feelings of belonging and the impact of overarching power dynamics in graduate education.

## 9. Implications

### 9.1. Implications for researchers

The power dynamics and expectations built into academia and STEM cultures have a profound impact on the student experience, particularly for neurodivergent and other marginalized students. The fact that many of the participants in this study explicitly mentioned the perception that their advisor holds power over their career and academic success suggests that the power dynamics involved in the advisor-advisee relationship may contribute to multiple challenges experienced by neurodivergent students. In particular, these dynamics may contribute to the difficulty that these students had with setting boundaries that would allow them to prioritize their own personal lives, mental health, and overall wellbeing. Additional research may be warranted to examine the impact of power dynamics within the advisor-advisee relationship.

It is important to note the disproportionate representation of women in this study. While we can only speculate about the reasons for this high level of representation, we suggest that this may be a meaningful data point. The most simple explanation may be that women who identify as neurodivergent are more likely to respond to recruitment emails than men who identify as such, or that women are more interested in talking about their experiences. However, it is also possible that the challenges faced by graduate students at the intersection of neurodiversity and gender may be amplified due to multiple, interacting layers of oppression, and that this in turn motivated more women to participate in research aimed at improving the educational environment. Additional research is needed to further explore the unique experiences of neurodivergent women and those with multiple marginalized identities in STEM fields.

### 9.2. Implications for practice

Graduate programs may also consider offering professional development to faculty advisors who work with graduate students to increase awareness of the strengths and challenges of neurodivergent students, to challenge the overarching norms and assumptions embedded in the graduate school experience and build more open pathways of communication. In particular, it is important to create an environment where there is open dialog between students and advisors, and more importantly, to break the stigma associated with discussing mental health, so that students feel comfortable coming forward and seeking needed supports. One feature that may be key in providing this type of environment is the adoption of a strengths-based approach toward neurodiversity that challenges the predominant deficit-based narrative toward neurological diversity and empowers neurodivergent students to leverage their strengths in the academic and research environment.

Practitioners may draw on recent scholarship that highlights the growing recognition of neurodiversity as an essential aspect of human diversity and emphasizes the importance of a strengths-based framework for neurodiversity in higher education. For example, Shmulsky et al. (2022) apply the principles of culturally relevant pedagogy (Ladson-Billings, 1995) to create inclusive learning environments for neurodivergent students, emphasizing the need to maintain high expectations, demonstrate acceptance of neurodiversity, and encourage the development of critical consciousness to better understand the power dynamics and social forces that shape the experiences of neurodivergent students. Similarly, Hamilton and Petty (2023) draw on psychological practices to outline a compassionate pedagogy for neurodiversity in higher education that emphasizes the development of educator empathy for neurodivergent learners’ experiences and prioritizes building personalized learning environments through a Universal Design for Learning (UDL) framework (CAST, 2018) to empower students to leverage their strengths in the classroom. If neurodivergent students feel that their strengths are valued, they may be more likely to build positive relationships based on honest communication about their experiences. By creating an environment in which students may “remove the mask,” programs may reduce the cognitive and emotional burden carried by neurodivergent students who are working hard to make it in graduate programs that were not designed for them.

Given the great importance of maintaining an advisor’s approval in order for students to succeed in graduate school and their career, efforts should be made by advisors and graduate school administrators to improve the quality of this relationship. This is especially important when considering the experiences of neurodivergent students, who may be more vulnerable to these power dynamics than their neurotypical peers. As previously noted, the advisor-advisee relationship falls outside of the realm of academic accommodations. While flexibility or accommodations from the advisor may assist students in some ways, we suggest that taking an accommodations approach to supporting neurodivergent students maintains an emphasis on student deficits and limits the potential of neurodivergent students to make unique contributions in their field because it fails to recognize and cultivate their unique strengths and talents. Rather, attention should be placed on enhancing the inclusivity of the culture within STEM programs and emphasizing the value of neurodiversity within academia to encourage innovation by leveraging nontraditional approaches to complex problems.

There are also implications for program administrators who shape policies and structures that are aimed at upholding rigorous standards but may have the unintended consequence of contributing to high stress levels, exhaustion and burnout among neurodivergent students. As the students in this study experienced much of their graduate program during the COVID-19 pandemic, some noted that their program had made specific moves toward more flexibility to accommodate and support student success and mental health during the pandemic. However, as the pandemic waned, they found themselves in an increasingly rigid environment. As programs have sought to “return to normal,” many have begun to reduce the flexibility that was made available during the COVID pandemic. Neurodivergent students who have benefited from programmatic changes such as extended deadlines and additional flexibility related to test-taking, now find themselves in an increasingly stressful and unaccommodating environment. Program leaders may consider how building in flexibility for students may support mental health and academic success for neurodivergent students.

## 10. Conclusion

This qualitative study used thematic analysis to examine the unique experiences of 18 neurodivergent students in graduate STEM programs at a large, R1 university. This paper focuses on the unique challenges they face, such as the invisibility of their neurological diversity, stigma, and pressure to mask their neurodivergent traits to fit in with their neurotypical peers. The findings from this study suggested a model of understanding the neurodivergent graduate student experience that is centered around the core experience of invisibility of neurodiversity and nested within the hierarchical structures, power dynamics, and assumptions embedded in STEM fields in higher education. Neurodivergent graduate students shoulder a heavy cognitive and emotional load as they strive to meet the norms of the neurotypical majority, hide their challenges and mask their neurodivergence, and silence themselves in the face of potentially threatening relationships. Even so, neurodivergent graduate students often possess a keen self-awareness that includes an appreciation of their unique perspectives, strengths, and thinking styles that they perceive may be a benefit to their STEM field. The findings highlighted challenging power dynamics within the advisor-advisee relationship that impede neurodivergent graduate students’ willingness to communicate with their advisors about their unique experiences, strengths and challenges.

## Author’s note

We prefer to use person-first language, as we believe it aligns most closely with a holistic approach that takes into account both the strengths and challenges of individuals. However, as a preference for identity-first language has been noted in the autism community, we have adopted this construction when referring to those on the autism spectrum. To reflect both of these approaches, and to preserve flow within the narrative, we have used both “neurodivergent students” and “students who identify as neurodivergent” within this paper.

## Data availability statement

The original contributions presented in the study are included in the article/supplementary material, further inquiries can be directed to the corresponding author.

## Ethics statement

The studies involving human participants were reviewed and approved by University of Connecticut Institutional Review Board (IRB). The patients/participants provided their written informed consent to participate in this study.

## Author contributions

AZ and RG designed the study. CS collected the data and performed the coding and analysis along with AH, with support and guidance from AZ, RG, and CB, and drafted all other sections with input from all authors. AH drafted the introduction and literature review. All authors contributed to the article and approved the submitted version.

## Funding

This material is based upon work supported by the National Science Foundation under NRT:IGE Grant No. 2105721. Any opinions, findings, and conclusions or recommendations expressed in this material are those of the author(s) and do not necessarily reflect the views of the National Science Foundation.

## Conflict of interest

The authors declare that the research was conducted in the absence of any commercial or financial relationships that could be construed as a potential conflict of interest.

## Publisher’s note

All claims expressed in this article are solely those of the authors and do not necessarily represent those of their affiliated organizations, or those of the publisher, the editors and the reviewers. Any product that may be evaluated in this article, or claim that may be made by its manufacturer, is not guaranteed or endorsed by the publisher.
